# Prevalence of Joint Gait Patterns Defined by a Delphi Consensus Study Is Related to Gross Motor Function, Topographical Classification, Weakness, and Spasticity, in Children with Cerebral Palsy

**DOI:** 10.3389/fnhum.2017.00185

**Published:** 2017-04-12

**Authors:** Angela Nieuwenhuys, Eirini Papageorgiou, Simon-Henri Schless, Tinne De Laet, Guy Molenaers, Kaat Desloovere

**Affiliations:** ^1^Neuromotor Research Group, Department of Rehabilitation Sciences, KU LeuvenLeuven, Belgium; ^2^Faculty of Engineering Science, KU LeuvenLeuven, Belgium; ^3^Department of Development and Regeneration, KU LeuvenLeuven, Belgium; ^4^Department of Orthopedics, University Hospitals LeuvenLeuven, Belgium; ^5^Clinical Motion Analysis Laboratory, University Hospitals LeuvenLeuven, Belgium

**Keywords:** cerebral palsy, gait, gait patterns, classification, prevalence, chi-square test

## Abstract

During a Delphi consensus study, a new joint gait classification system was developed for children with cerebral palsy (CP). This system, whose reliability and content validity have previously been established, identified 49 distinct joint patterns. The present study aims to provide a first insight toward the construct validity and clinical relevance of this classification system. The retrospective sample of convenience consisted of 286 patients with spastic CP (3–18 years old, GMFCS levels I–III, 166 with bilateral CP). Kinematic and kinetic trials from three-dimensional gait analysis were classified according to the definitions of the Delphi study, and one classified trial was randomly selected for each included limb (*n* = 446). Muscle weakness and spasticity were assessed for different muscle groups acting around the hip, knee, and ankle. Subsequently, Pearson Chi square tests, Cramer's V, and adjusted standardized residuals were calculated to explore the strength and direction of the associations between the joint patterns, and the different patient-specific characteristics (i.e., age, GMFCS level, and topographical classification) or clinical symptoms (muscle weakness and spasticity). Patient-specific characteristics showed several significant associations with the patterns of different joints, but the strength of most identified associations was weak. Apart from the knee during stance phase and the pelvis in the sagittal plane, the results systematically showed that the patterns with “minor gait deviations” were the most frequently observed. These minor deviations were found significantly more often in limbs with a lower level of spasticity and good muscle strength. Several other pathological joint patterns were moderately associated with weakness or spasticity, including but not limited to “outtoeing” for weakness and “intoeing” for spasticity. For the joints in the sagittal plane, significantly stronger associations were found with muscle weakness and spasticity, possibly because most of the evaluated muscles in this study mainly perform sagittal plane motions. Remarkably, the hip patterns in the coronal plane did not associate significantly with any of the investigated variables. Although further validation is warranted, this study contributes to the construct validity of the joint patterns of the Delphi consensus study, by demonstrating their ability to distinguish between clinically relevant subgroups in CP.

## Introduction

Cerebral palsy (CP) is the result of a pre- or post-natal lesion in the developing brain of a fetus or child, primarily affecting motor behavior. The heterogenic clinical presentation of CP is emphasized, not only because of the numerous potential differences in timing, location, severity, and nature of brain lesions, but also because it is continuously altered by a maturing brain, musculoskeletal growth, and treatment (Bax et al., 2005). For epidemiological, treatment-related, and many other reasons, it is therefore important to identify relevant subgroups within the CP population. Several important categorizations of subgroups in CP have been reported before. For instance, the Gross Motor Function Classification System (GMFCS) and the Manual Ability Classification System are used to classify the severity of lower and upper limb motor function impairment (Palisano et al., 1997; Eliasson et al., 2006), while emphasizing on everyday performance (Palisano et al., 1997). Because of the complex interaction between primary and secondary motor symptoms in CP, for example between spasticity and muscle contractures, gait pathology varies a lot between patients. Hence, even though the GMFCS is a generally accepted functionality score for children with CP, it is not detailed enough to cover all gait-related deviations (Õunpuu et al., 2015).

In literature, several gait classifications have been defined based on three-dimensional gait analysis data (i.e., kinematics, kinetics, or muscle activation data) (Vaughan and O'Malley, 2005; Dobson et al., 2007; Toro et al., 2007; Ferrari et al., 2008; Carriero et al., 2009; Rozumalski and Schwartz, 2009; Bonnefoy-Mazure et al., 2013; Davids and Bagley, 2014). Gait classifications aim to define groups of gait deviations into distinct categories and may be built based on either qualitative or quantitative methods (Dobson et al., 2007). Recently, a new, qualitative overview of joint patterns during gait for all ambulatory children with spastic CP has been described, covering the wide range of gait deviations in the relevant lower limb joints across the three anatomical planes (Nieuwenhuys et al., 2016). Through a Delphi consensus study, an international expert panel defined 49 joint patterns during gait. Separate patterns were defined for the pelvis, hip, knee, ankle, and foot in the sagittal, coronal, and transverse planes. Recently, the content validity of this classification system (Nieuwenhuys et al., 2017a) was investigated on a cohort of 356 patients with CP and 56 typically developing (TD) children. Two experienced raters classified more than 1,700 kinematic and kinetic trials. Subsequently, the mean kinematic and kinetic waveforms for each pattern and the pattern of TD children were analyzed using statistical parametric mapping (SPM) (Pataky, 2010) to verify (1) whether the existence of the patterns and the subjective rules, which were defined during the consensus study, could be confirmed and (2) whether potential patterns and relevant information might have been missed. The results indicated that for each pattern, all key locations that were included in the pattern definitions, were also indicated as significant areas by the SPM analysis. A detailed definition of the different joint patterns is provided in Table S1 in the Supplementary Material. As the previously mentioned content validity study highlighted, the patterns that were originally labeled as “normal” may be misleading (Nieuwenhuys et al., 2017a). Hence, in Table S1, the original definitions of these joint patterns were modified to “minor gait deviations.” Previous research has also showed that the created classification can be reliably used, even by inexperienced clinicians, displaying reliability levels that ranged between “substantial” to “almost perfect agreement” for all joints, except for the knee patterns during stance phase that showed moderate agreement (Nieuwenhuys et al., 2017b). However, the construct validity of this newly introduced joint gait classification system and its relevance for clinical and research practice has not yet been examined.

The construct validity can be assessed, by comparing the gait classification with a criterion classification (Zwick et al., 2004), or by assessing its relationships with scores of other instruments. Previous research has already shown the relevance of establishing the relation between specific gait features and other variables such as topographical classification, age, preceding treatments, and clinical measurements (Wren et al., 2005; Domagalska et al., 2013). Further, Rozumalski et al. (Rozumalski and Schwartz, 2009) investigated how different crouch gait patterns, which were determined via k-means cluster analysis, were characterized by range of motion, muscle strength, and spasticity. Dobson et al. (2011) reported on the construct validity of the Winters classification, by showing how the distribution of the patterns was associated with other validated classifications such as the Gross Motor Function Classification Scale (Palisano et al., 1997) (GMFCS) and Functional Mobility Scale (Graham et al., 2004). By providing evidence that the classification can make a distinction between relevant subgroups in CP, its usefulness and validity can be demonstrated.

The present study aims to provide a first insight toward the construct validity and clinical relevance of the aforementioned consensus-based joint patterns during gait in children with CP (Nieuwenhuys et al., 2016). The prevalence of the patterns and their association with other patient-specific characteristics and clinical symptoms, in particular muscle weakness and spasticity, is explored in an extended patient cohort. It is hypothesized that the prevalence of the patterns is associated with age, topographical classification, GMFCS level, and previous treatment. The study also examines how specific joint patterns are characterized by weakness and spasticity. It is hypothesized that pelvis and hip patterns are associated in particular with the severity of weakness or spasticity in muscle groups that have a function around the pelvis and hip joint. Analogous to the previous hypothesis, knee and ankle patterns are expected to associate with the presence of weakness or spasticity in the muscles acting at the knee and ankle respectively.

## Materials and methods

### Patient recruitment

This study was approved by the Medical Ethical Committee of University Hospitals Leuven (s56036). An extended retrospective convenience sample was available from the database of the hospital, comprising gait analysis sessions that were obtained for research or clinical purposes between November 2001 and August 2015. The sample contained a total of 459 sessions (from 356 children), which were all screened for the following inclusion criteria: (a) a diagnosis of unilateral or bilateral CP (b) predominantly spastic type of CP (c) 3–18 years of age, (d) GMFCS-level I–III, and (e) the availability of at least two good quality kinematic gait trials from three-dimensional gait analysis.

### Instrumented gait analysis

Standardized three-dimensional gait analyses were performed using 10 to 15 VICON motion camera's (Vicon Motion Systems, Oxford, UK) and two AMTI force plates (Advanced Mechanical Technology Inc., Watertown, MA, USA). Reflecting markers were placed on anatomical landmarks of the patient according to the Plug-In-Gait marker model and patients were instructed to walk barefoot and at a self-selected speed on a 10 m-walkway. Nexus software was used to define gait cycles and to estimate joint angles and joint moments in the three anatomical planes. For each kinematic and kinetic trial, one step per side (left and right) was identified. For patients with unilateral CP, only the affected body side was selected for all analyses. For patients with bilateral involvement, both sides were included in the analyses of side-specific variables (i.e., “previous surgery,” spasticity, and weakness scores), while for all other comparisons, one side was randomly selected. All available steps were visually screened and steps with artifacts, signs of inaccurate marker placement, or steps that were not representative of a patient's gait (outliers), were excluded so that only trials of good quality remained. The remaining good quality steps, 1,719 in total, were then classified by a clinician who was experienced with the joint patterns (AN or EP). As a result, for each gait analysis session, one to seven steps per side per patient were classified. Subsequently, for each included session, one classified step was randomly selected per side, unless a pattern with a very low prevalence in the database was present (< 10% of 1,719 trials), in which case that step was given priority. In a previous study, the reliability with which both raters could identify the joint patterns was assessed using a sample of 82 children with CP. Interrater agreement was shown to be almost perfect (overall percentage of agreement = 90%, kappa = 0.86, confidence interval = 078–0.94). Table 1 shows the prevalence of the joint patterns in the recruited sample as well as a concise description of the patterns per joint.

**Table 1:** **Brief definition of all joint patterns during gait and their prevalence in the selected limbs (***N*** = 446) from the patient population**.

**Sagittal plane**	***N* (%)**
**PELVIS**	
PS0—Minor gait deviations	88 (19.7)
PS1—Increased range of motion	130 (29.1)
PS2—Increased anterior tilt on average	67 (15.0)
PS3—Increased anterior tilt and increased range of motion	157 (35.2)
PS4—Decreased anterior tilt (posterior tilt)	1 (0.2)
PS5—Decreased anterior tilt (posterior tilt) and increased range of motion	3 (0.7)
**HIP**	
HS0—Minor gait deviations	229 (51.3)
HS1—Hip extension deficit	136 (30.5)
HS2—Continuous excessive hip flexion	81 (18.2)
**KNEE DURING STANCE**	
KSTS0—Minor gait deviations	56 (12.6)
KSTS1—Increased knee flexion at initial contact	33 (7.4)
KSTS2—Increased knee flexion at initial contact and earlier knee extension movement	89 (20.0)
KSTS3—Knee hyperextension	38 (8.5)
KSTS4—Knee hyperextension and increased knee flexion at initial contact	53 (11.9)
KSTS5—Increased flexion in midstance and internal flexion moment present	100 (22.4)
KSTS6—Increased flexion in midstance and internal extension moment present	77 (17.3)
**KNEE DURING SWING**	
KSWS0—Minor gait deviations	140 (31.4)
KSWS1—Delayed peak knee flexion	103 (23.1)
KSWS2—Increased peak knee flexion	50 (11.2)
KSWS3—Increased and delayed peak knee flexion	42 (9.4)
KSWS4—Decreased peak knee flexion	53 (11.9)
KSWS5—Decreased and delayed peak knee flexion	58 (13.0)
**ANKLE DURING STANCE**	
ASTS0—Minor gait deviations	164 (36.8)
ASTS1—Horizontal second ankle rocker	133 (29.8)
ASTS2—Reversed second ankle rocker	53 (11.9)
ASTS3—Equinus gait	22 (4.9)
ASTS4—Calcaneus gait	74 (16.6)
**ANKLE DURING SWING**	
ASWS0—Minor gait deviations	165 (37.0)
ASWS1—Insufficient prepositioning in terminal swing	39 (8.7)
ASWS2—Continuous plantarflexion during swing (drop foot)	94 (21.1)
ASWS3—Excessive dorsiflexion during swing	148 (33.2)
**Coronal plane**	***N*** **(%)**
**PELVIS**	
PC0—Minor gait deviations	225 (50.4)
PC1—Increased pelvic range of motion	135 (30.3)
PC2—Continuous pelvic elevation	34 (7.6)
PC3—Continuous pelvic depression	52 (11.7)
**HIP**	
HC0—Minor gait deviations	278 (62.3)
HC1—Excessive hip abduction in swing	87 (19.5)
HC2—Continuous excessive hip abduction	52 (11.7)
HC3—Continuous excessive hip adduction	29 (6.5)
**Transverse plane**	
**PELVIS**	
PT0—Minor gait deviations	204 (45.7)
PT1—Increased pelvic range of motion	136 (30.5)
PT2—Excessive pelvic external rotation during the gait cycle	66 (14.8)
PT3—Excessive pelvic internal rotation during the gait cycle	40 (9.0)
**HIP**	
HT0—Minor gait deviations	338 (75.8)
HT1—Excessive hip external rotation during the gait cycle	34 (7.6)
HT2—Excessive hip internal rotation during the gait cycle	74 (16.6)
**FOOT PROGRESSION ANGLE**	
FPA0—Minor gait deviations	279 (62.6)
FPA1—Outtoeing	73 (16.4)
FPA2—Intoeing	94 (21.1)

*Definitions of the joint patterns are provided in Table S1 (Supplementary Material). Described deviations such as increased or excessive joint angles refer to deviations which are more than one standard deviation away from a reference database of typically developing children. A more detailed description of the patterns is available in Nieuwenhuys et al. (2016)*.

One gait analysis session was selected for each patient. Sessions were excluded if a patient had undergone Botulinum toxin type A treatment less than 180 days or surgery (i.e., single event multilevel surgery or selective dorsal rhizotomy) less than 365 days before the date of the gait analysis session. In case more than one session was still available for a patient, preference was given to the earliest pre-treatment session with the least amount of missing data from the clinical examination.

### Clinical examination of weakness and spasticity

Gait analysis sessions were preceded by a clinical examination during which muscle strength and muscle tone were evaluated. Isometric muscle strength was assessed by experienced physiotherapists using the manual muscle testing scale (MMT) (Daniels and Worthingham, 1986; Cuthbert and Goodheart, 2007). The MMT is scored on a six-point ordinal scale (scores range from 0 to 5) and it differentiates between a palpable contraction and a motion against gravity or against resistance. The maximum score of 5 indicates that a patient can move for the full range of motion against gravity and maximum resistance, whereas a score of 0 indicates that no contraction can be palpated. Isometric strength was assessed and scored for the following muscle groups: hip flexors, extensors, adductors, and abductors; knee flexors and extensors; ankle dorsi- and plantar flexors, and the muscle groups performing ankle inversion and eversion. In addition, muscle spasticity was evaluated using the Modified Ashworth Scale (MAS) (Mutlu et al., 2008), which is also a six-point ordinal scale (scores: 0, 1, 1+, 2, 3, 4), The MAS classifies the extent of increase in muscle tone felt by the assesor during the stretch of a passive muscle group through the full range of motion. The maximum score of 4 indicates that the evaluated muscle or muscle group is rigid and no motion is possible, whereas a score of 0 indicates a normal muscle tone. MAS scores were collected for the hip flexors, short adductors, and long adductors; for the hamstrings and rectus femoris muscles at the level of the knee; and for the gastrocnemius, soleus, and tibialis posterior muscles at the level of the ankle joint.

Because of the high number of muscles that were evaluated during the clinical examination and because of the explorative nature of the study, it was decided to group the muscles according to the joints around which they have their main function, such that the hip, knee, and ankle joint were characterized by one score for muscle weakness and one score for spasticity. For instance, the highest MAS score between the gastrocnemius, soleus, and tibialis posterior muscles was selected to represent the severity of spasticity around the ankle joint. The involved multidisciplinary team advised to select the most severe score for weakness (i.e., lowest score) and spasticity (i.e., highest score) at the level of each joint because of two reasons: on the one hand, the muscles most affected by weakness or spasticity were considered to have a larger influence on pathological gait deviations. On the other hand, the selection of the most severe score per joint, instead of averaged values or summation of muscle-specific scores, ensured that the impact of weakness or spasticity would not be filtered out (which might be expected if the average of the joint sub-scores was used). In addition, the clinical examination data was characterized by missing data as a result of the retrospective nature of the study. By selecting the most severe score per joint, the sample size of the study would not be reduced, which was expected to happen if the muscle-specific scores were summed. The influence of these missing data on the results was expected to be negligible, as the median percentage of missing data per MAS or MMT variable was 0.44% (range 0–5.6%). To illustrate the associations between the defined joint gait patterns and muscle weakness and spasticity, clinical case examples of the kinematic waveforms in combination with the respective scores of MAS or MMT, and supported by video fragments, are presented in Supplementary Material—Video 1.

### Statistical analysis

The first level of construct validity was evaluated by studying the association of the joint patterns with age, GMFCS level, previous orthopedic surgery and topographical classification (unilateral vs. bilateral CP). While the GMFCS cannot be considered detailed enough to report on specific gait-related deviations, it is a clinically accepted score of overall functionality of CP children. Therefore, it has been used to establish a relation between the severity of pathological function and the occurrence of each joint pattern. The next level of construct validity was evaluated by studying the association of the joint patterns with clinical examination scores (i.e., weakness of the muscles around the hip, knee, and ankle; spasticity of the muscles around the hip, knee, and ankle).

Descriptive statistics and cross-tables were used to describe the frequency distributions for all patterns, as well as for the patient-specific characteristics and clinical symptoms. Age was further categorized into three groups using the 25 and 75th percentile as cut-off values. These categories will further be referred to as the “youngest patients” (patients until 7.5 years old), “medium aged patients” (patients from 7.5 to 12.5 years old), and “oldest patients” (patients over 12.5 years old).

Pearson Chi-square tests (χ^2^) were performed to investigate if the distribution of the patient-specific characteristics and clinical symptoms were significantly associated with the distribution of the patterns at the level of each joint (α = 0.05). In χ^2^, observed frequencies of individual counts are compared to expected frequencies which would be expected by chance. To allow for a valid interpretation of χ^2^, a sufficiently large sample size is required for all the associations that were tested between the patient-specific characteristics or clinical symptoms (weakness and spasticity) and the joint patterns. Following the principles of χ^2^ (Portney and Watkins, 2009), the expected frequency of cells should be at least *n* = 1 for every parameter and the expected frequencies below *n* = 5 can only be accepted in less than 20% of the cells of the cross-tables (Portney and Watkins, 2009). When this condition was not met and expected frequencies were less than *n* = 5 for a specific variable, two categories of a variable were combined, however only when merging those categories was clinically meaningful (e.g., Scores 4 and 5 of the MMT were often combined, both scores indicating that the patient could move against moderate to heavy resistance). In case of significant associations, the strength of the association was evaluated using Cramer's V, which is dependent on the degrees of freedom (DF). The DF was defined by the smallest value of the data (either rows or columns). For example, when examining the association between uni- or bilateral CP and the patterns of the hip in the sagittal plane, the DF were defined by the uni-/bilateral distribution and not by the hip patterns (*n* = 2 and *n* = 3 respectively). The strength of the association based on Cramer's V was thereby classified as weak, moderate or strong (Table S2) (Cohen, 1988). Subsequently, adjusted standardized residuals (ASR) were examined to explore the direction of significant associations. ASRs can identify significant combinations of specific categories of two variables that contributed stronger to the identified association than other combinations of categories. Because ASRs follow a normal distribution with mean “0” and standard deviation “1,” ASR values larger than –2 or +2 indicate that the frequency count in a particular cell is respectively significantly smaller or higher than would be expected if the two variables were unrelated (*p* < 0.05).

## Results

### Description of experimental patient population

After the data selection process, the experimental sample consisted of 286 patients with spastic CP of which the majority had a diagnosis of bilateral CP (*n* = 166) and the median age was 10.2 years (Table 2). Gait analysis sessions of patients who had undergone previous orthopedic surgery were collected after a median of ~2 years (interquartile range: 1 year and 3 months—5 years and 6 months). Because both sides could be included for the majority of the patients with bilateral CP, a total of 446 limbs were used for the statistical analyses of side-specific variables (i.e., “previous surgery,” spasticity, and weakness scores).

**Table 2:** **Patient characteristics (***N*** = 286)**.

	***N* (%)**	
**GENDER**		
Male	165 (57.7)	
Female	121 (42.3)	
**DIAGNOSIS**		
Bilateral CP	166 (58.0)	
Unilateral CP	120 (42.0)	
**GMFCS**		
Level I	172 (60.1)	
Level II	89 (31.1)	
Level III	25 (8.7)	
**PREVIOUS ORTHOPEDIC SURGERY**		
Yes	55 (19.2)	(*n* = 100 limbs)
No	231 (80.8)	(*n* = 346 limbs)
**NUMBER OF PREVIOUS BOTULINUM TOXIN TYPE A TREATMENTS**		
None	111 (38.8)	(*n* = 159 limbs)
One or two	104 (36.4)	(*n* = 155 limbs)
Three or more	71 (24.8)	(*n* = 132 limbs)
Weight [mean (*SD*), in kg]	34.3 (14.8)	
Height [mean (*SD*), in cm]	137.6 (19.7)	
Age at time of gait analysis [median (IQR), in years]	10.2 (7.5-12.5)	

*SD, standard deviation; IQR, interquartile range*.

Table 3 presents the frequency distribution of the spasticity and weakness scores around the hip, knee, and ankle joint, representing the sum of the most severe scores per joint (see section Clinical examination of weakness and spasticity). The muscles acting around the hip were least affected by spasticity, with 48.5% of all limbs classified as MAS 0 or 1. On the contrary, muscles around the ankle joint were most severely affected by spasticity, with 42.7% of all limbs classified as MAS 2, 3, or 4. The weakest muscle groups were also those with their main function around the ankle, with 16.3% of all limbs classified as MMT 0 or 1 as opposed to 1.6% and 0% for the same MMT scores at the hip and knee joint.

**Table 3:** **Prevalence and distribution of MAS and MMT scores for the muscles around the hip, knee, and ankle joint in the selected limbs (***N*** = 446) from the patient population**.

	**MAS score [*****N*** **(%)]**					
	**0**	**1**	**1+**	**2**	**3**	**4**
Hip	93 (20.9)	123 (27.6)	130 (29.1)	98 (22.0)	2 (0.4)	0 (0.0)
Knee	22 (4.9)	118 (26.5)	153 (34.3)	142 (31.8)	11 (2.5)	0 (0.0)
Ankle	9 (2.0)	46 (10.3)	196 (43.9)	164 (36.8)	26 (5.8)	5 (1.1)
	**MMT score [*****N*** **(%)]**					
	**0**	**1**	**2**	**3**	**4**	**5**
Hip	0 (0.0)	7 (1.6)	33 (7.4)	231 (51.8)	162 (36.3)	13 (2.9)
Knee	0 (0.0)	0 (0.0)	14 (3.1)	191 (42.8)	221 (49.6)	20 (4.5)
Ankle	5 (1.1)	68 (15.2)	85 (19.1)	189 (42.4)	83 (18.6)	16 (3.6)

*The muscles around the hip, knee, and ankle joint are summed following the approach described in section Clinical examination of weakness and spasticity. If less than 50 limbs were classified in a particular category of the MMT or MAS scale, the expected frequencies in the cross-tables were generally too low to allow a valid interpretation of χ^2^, especially for analyses in combination with joints that have a high number of patterns [e.g., knee during stance (n = 7)]. Therefore, darker shaded categories were merged at the level of each joint, all indicating a lower level of spasticity or a higher level of muscle weakness. Lightly shaded areas were merged at the level of each joint, indicating a higher level of spasticity and a lower level of muscle weakness*.

Table 1 presents the prevalence of the 49 patterns. Except for the knee during stance and the pelvis in the sagittal plane, the pattern with “minor gait deviations” was the most prevalent one in all other joints, indicating that patients mostly remained within one standard deviation from the mean of an age-matched group of typically developing children. Pathological patterns that were observed most frequently in the proximal joints were “increased pelvic anterior tilt and increased range of motion” (35.2%), “hip extension deficit” (30.5%), and “increased pelvic range of motion” in the sagittal (29.1%), coronal (30.3%), and transverse (30.5%) plane. For the distal joints, the patterns “excessive ankle dorsiflexion during swing” (33.2%), “horizontal second ankle rocker during stance” (29.8%), “delayed peak knee flexion during swing” (23.1%), and “excessive knee flexion and internal flexion moment during stance” (22.4%) were most frequently observed. Because the prevalence of “decreased pelvic anterior tilt” (0.2%) and “decreased pelvic anterior tilt and increased range of motion” (0.7%) was extremely low, both patterns needed to be excluded from further statistical analyses.

**Figure 1:**
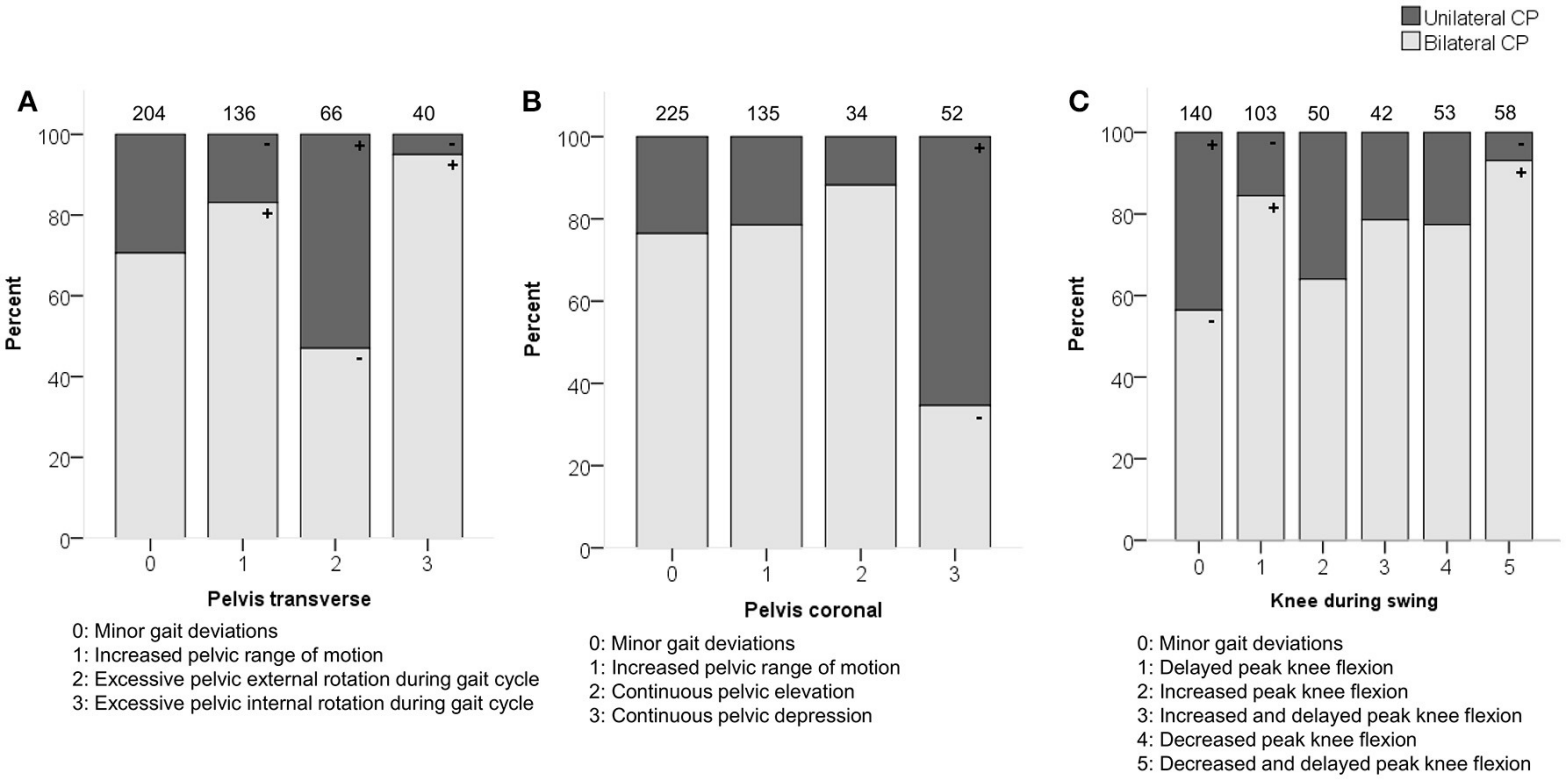
**Topographical classification associated moderately with (A)** pelvis patterns in transverse plane (PT) **(B)** pelvis patterns in coronal plane (PC) and **(C)** knee patterns during swing (KSWS). The symbol “+” indicates that a pattern was observed significantly more frequently and “–” indicates that a pattern was observed significantly less frequently in children with unilateral or bilateral CP (*p* < 0.05). Specific ASRs are available in Tables S4, S6, S7. Numbers on top of each bar represent the number of patients that were classified into that pattern.

**Figure 2:**
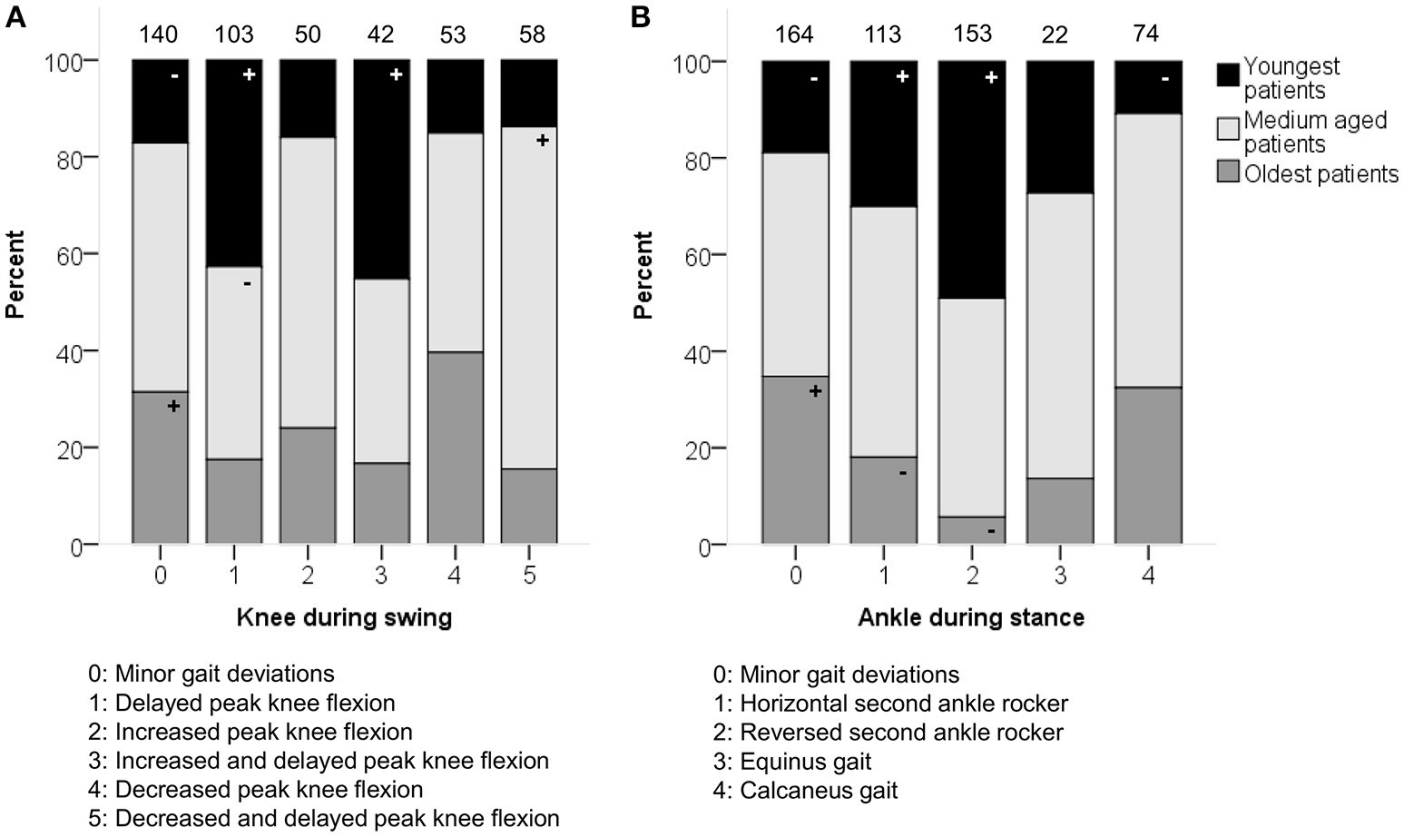
**Age associated moderately with the distribution of (A)** knee patterns during swing (KSWS) and **(B)** ankle patterns during stance (ASTS). The symbol “+” indicates that a pattern was observed significantly more frequently and “–” indicates that a pattern was observed significantly less frequently in the youngest, medium aged, or oldest patients (*p* < 0.05). Specific ASRs are available in Tables S4, S5. Numbers on top of each bar represent the number of patients that were classified into that pattern.

**Figure 3:**
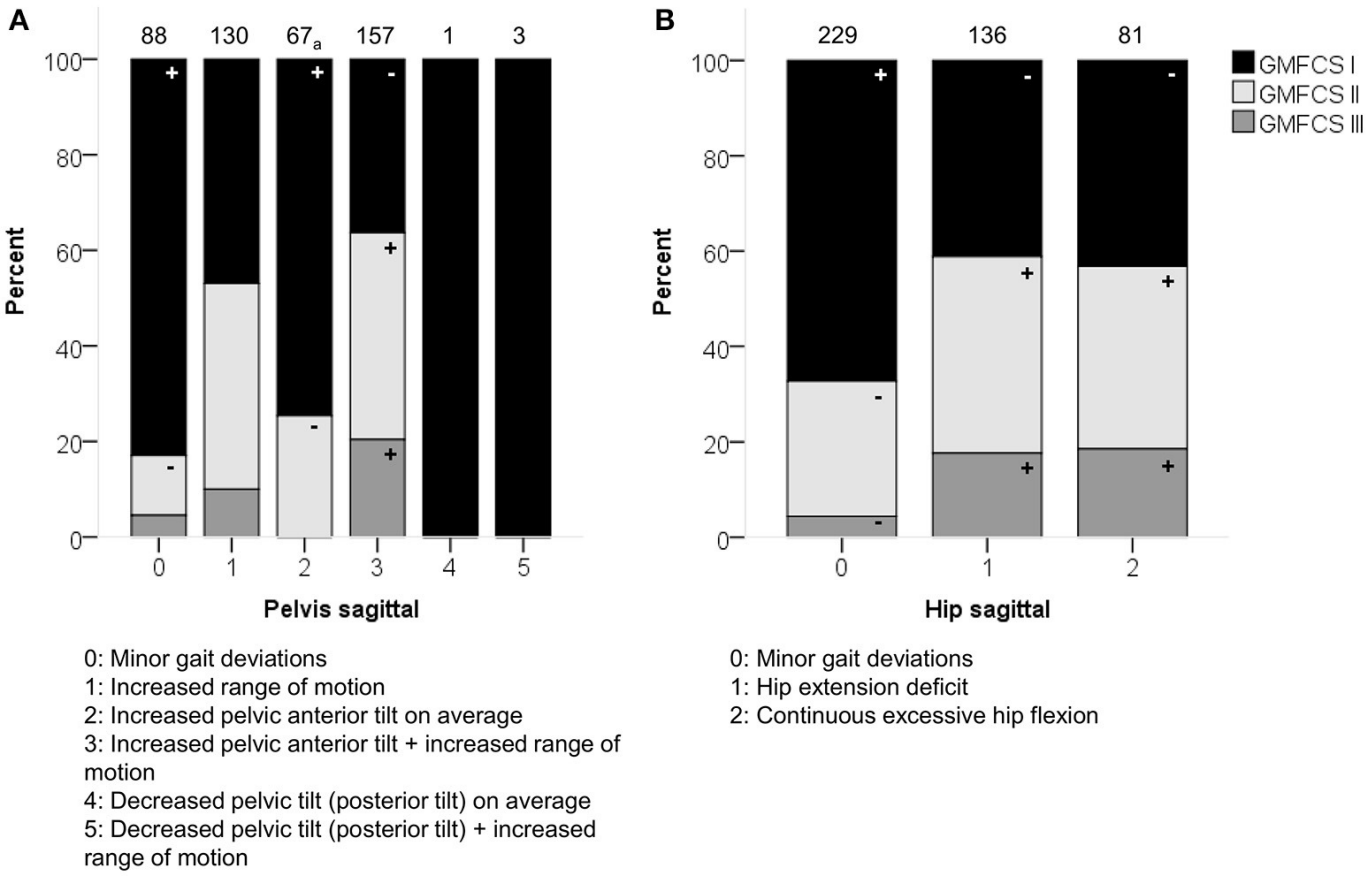
**GMFCS level associated moderately with the distribution of (A)** pelvis patterns in sagittal plane (PS) and **(B)** hip patterns in sagittal plane (HS). The symbol “+” indicates that a pattern was observed significantly more frequently and “–” indicates that a pattern was observed significantly less frequently in patients with GMFCS level I, II, or III (*p* < 0.05). ^a^Indicates that increased pelvic anterior tilt (PS2) was observed significantly less often in patients with GMFCS III. Specific ASRs are available in Table S3. Numbers on top of each bar represent the number of patients that were classified into that pattern.

**Figure 4:**
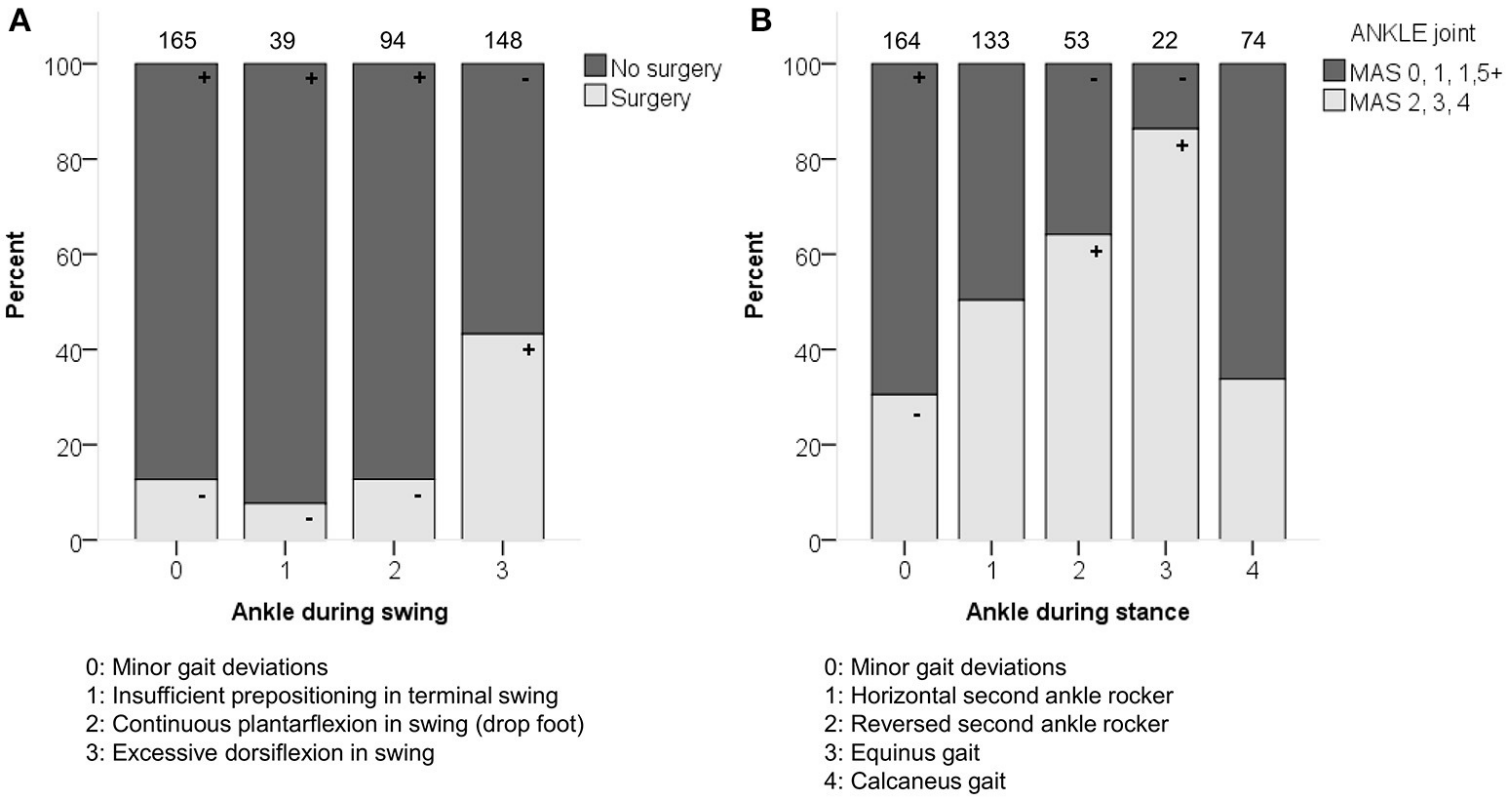
**(A)** Previous surgery associated moderately with the distribution of the ankle patterns during swing (ASWS). **(B)** Spasticity of muscles acting around the ankle associated moderately with the distribution of the ankle patterns during stance (ASTS). The symbol “+” indicates that a pattern was observed significantly more frequently and “–” indicates that a pattern was observed significantly less frequently in limbs with or without surgery, or in limbs with lower (MAS 0, 1, 1+) vs. higher (MAS 2, 3, 4) levels of spasticity around the ankle (*p* < 0.05). Specific ASRs are available in Table S5 and video illustrations of some joint gait patterns are available in Video 1. Numbers on top of each bar represent the number of limbs that were classified into that pattern.

**Figure 5:**
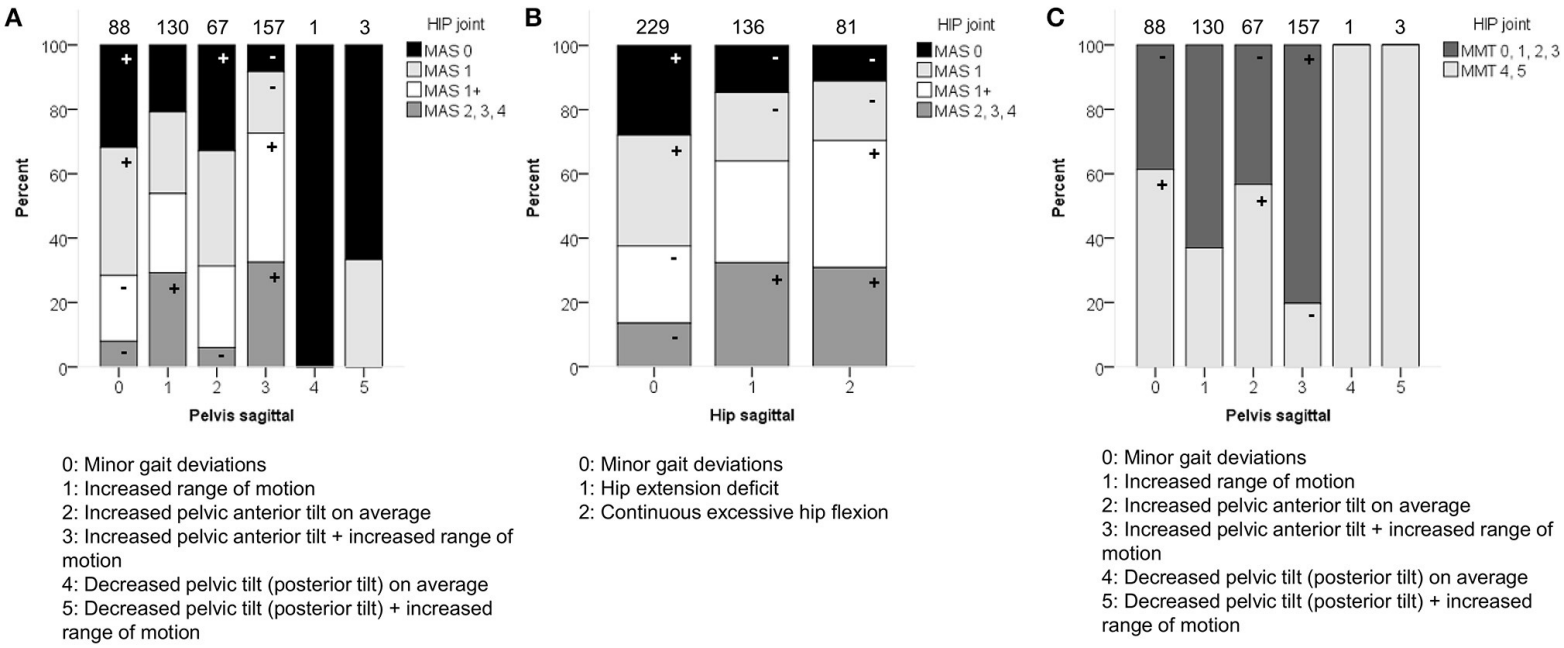
**Spasticity of muscles acting around the hip associated moderately with the distribution of (A)** pelvis patterns in sagittal plane (PS), and **(B)** hip patterns in sagittal plane (HS). **(C)** Weakness of muscles acting around the hip associated moderately with PS. The symbol “+” indicates that a pattern was observed significantly more frequently and “–” indicates that a pattern was observed significantly less frequently in limbs with of a particular MAS score or in limbs with weaker (MMT 0, 1, 2, 3) or stronger (MMT 4, 5) muscles around the hip (*p* < 0.05). Specific ASRs are available in Table S3 and video illustrations of some joint gait patterns are available in Video 1. Numbers on top of each bar represent the number of limbs that were classified into that pattern.

**Figure 6:**
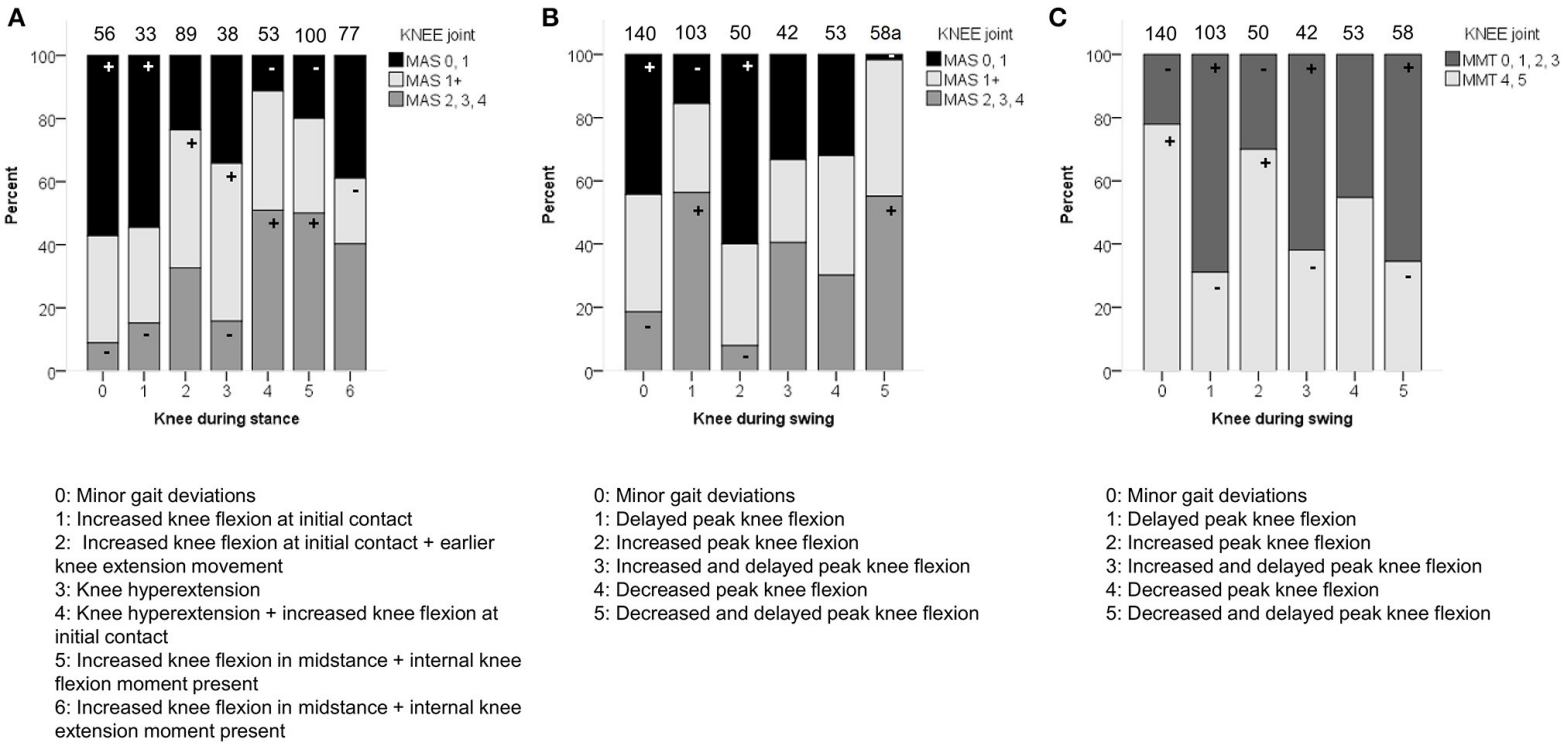
**Spasticity of muscles acting around the knee associated moderately with the distribution of (A)** knee patterns during stance (KSTS), and **(B)** knee patterns during swing (KSWS). **(C)** Weakness of muscles acting around the knee associated moderately with KSWS. The symbol “+” indicates that a pattern was observed significantly more frequently and “−” indicates that a pattern was observed significantly less frequently in limbs with of a particular MAS score or in limbs with weaker (MMT 0, 1, 2, 3) or stronger (MMT 4, 5) muscles around the knee (*p* < 0.05). ^a^Indicates that decreased and delayed peak knee flexion (KSWS5) was observed significantly less often with limbs classified as MAS 0 or 1. Specific ASRs are available in Table S4 and video illustrations of some joint gait patterns are available in Video 1. Numbers on top of each bar represent the number of limbs that were classified into that pattern.

Tables 4, 5 report the results of all χ^2^ analyses, which established the associations between the distribution of the joint patterns during gait and the patient-specific variables, previous surgery, spasticity, and weakness. Because many significant associations were identified, only the directions of significant moderate associations, where the ASR reached a value larger than 2, are discussed in detail (Figures 1–**6**). Detailed information on the direction of significant weak associations (ASRs) is available in Tables S3–S7 in the Supplementary Material.

**Table 4:** **Pearson chi squared analyses (χ^**2**^) and Cramer's V (***V***) identified significantly weak, moderate, and strong associations between the sagittal plane joint patterns and patient-specific characteristics, previous surgery, spasticity, and weakness**.

	**PS^b^**		**HS**		**KSTS**		**KSWS**		**ASTS**		**ASWS**	
	**χ^2^**	***V***	**χ^2^**	***V***	**χ^2^**	***V***	**χ^2^**	***V***	**χ^2^**	***V***	**χ^2^**	***V***
***N*** = **286 PATIENTS**												
Uni-/bilateral CP	7.77	0.17	8.84^*^	0.18	24.69^**^	0.29	27.46^***^	0.31	5.83	0.14	20.66^**^	0.27
Age	13.21^*^	0.15	11.03^*^	0.14	16.95	0.17	37.08^***^	0.26	28.02^**^	0.22	9.02	0.13
GMFCS	38.96^***^	0.26	30.49^***^	0.23	64.70^a^^***^	0.34	53.73^a^^***^	0.31	27.00^a^^*^	0.22	10.31	0.13
***N*** = **446 LIMBS**												
Previous surgery	8.26^*^	0.14	8.83^*^	0.14	14.40^*^	0.18	1.05	0.05	18.70^*^	0.21	55.71^***^	0.35
MAS Hip joint	68.51^***^	0.23	41.95^***^	0.22	81.37^***^	0.25	149.48^***^	0.33	44.60^***^	0.18	14.16	0.10
MAS Knee joint	44.23^***^	0.22	27.41^***^	0.18	71.86^***^	0.28	91.68^***^	0.32	29.64^**^	0.18	18.47^*^	0.14
MAS Ankle joint	29.12^***^	0.26	4.07	0.10	39.30^***^	0.30	67.69^***^	0.39	42.28^***^	0.31	17.20^*^	0.20
MMT Hip joint	52.18^***^	0.34	30.25^***^	0.26	48.80^***^	0.33	51.31^***^	0.34	9.35	0.15	12.82^*^	0.17
MMT Knee joint	57.67^***^	0.36	35.44^***^	0.28	36.51^***^	0.29	72.23^***^	0.40	18.91^*^	0.21	10.33^*^	0.15
MMT Ankle joint	79.96^***^	0.25	38.31^***^	0.21	59.66^***^	0.21	78.05^***^	0.24	28.85^*^	0.15	33.43^**^	0.16

* p < 0.05;

** p < 0.001;

*** p < 0.0001; χ^2^, Pearson chi squared; V, Cramer's V, indicating significantly weak (light gray), moderate (darker gray), and strong (dark gray) associations based on degrees of freedom (section Statistical analysis and Table S2);

a Results should be interpreted with caution because >20% of cells had expected frequencies lower than n = 5;

b *N = 282 patients and N = 442 limbs due to exclusion of PS4 and PS5*.

**Table 5:** **Pearson chi squared analyses (χ^**2**^) and Cramer's V (***V***) identified significantly weak and moderate associations between the coronal and transverse plane joint patterns and patient-specific characteristics, previous surgery, spasticity, and weakness**.

	**PC**		**HC**		**PT**		**HT**		**FT**	
	**χ^2^**	***V***	**χ^2^**	***V***	**χ^2^**	***V***	**χ^2^**	***V***	**χ^2^**	***V***
***N*** = **286**										
Uni-/bilateral CP	24.92^***^	0.30	2.42	0.09	26.49^***^	0.30	3.10	0.10	14.56^*^	0.23
Age	13.63^*^	0.15	4.89	0.09	4.43	0.09	2.88	0.07	11.46^*^	0.14
GMFCS	10.02	0.13	17.28^a^^*^	0.17	19.42^*^	0.18	12.71^a^^*^	0.15	7.60	0.12
***N*** = **446**										
Previous surgery	8.38^*^	0.14	2.29	0.07	2.71	0.08	10.25^*^	0.15	2.03	0.07
MAS Hip joint	23.84^*^	0.13	3.18	0.05	15.98	0.11	28.79^***^	0.18	21.75^*^	0.16
MAS Knee joint	19.51^*^	0.15	1.84	0.05	16.97^*^	0.14	15.31^*^	0.13	10.70^*^	0.11
MAS Ankle joint	6.32	0.12	5.24	0.11	5.07	0.11	8.94^*^	0.14	4.40	0.10
MMT Hip joint	12.64^*^	0.17	1.44	0.06	11.39^*^	0.16	9.31^*^	0.14	5.53	0.11
MMT Knee joint	9.26^*^	0.14	3.82	0.09	5.74	0.11	16.61^**^	0.19	7.42^*^	0.13
MMT Ankle joint	14.53	0.10	10.12	0.09	28.51^*^	0.15	23.61^*^	0.16	13.49^*^	0.12

* p < 0.05;

** p < 0.001;

*** p < 0.0001; χ^2^, Pearson chi squared; V, Cramer's V, indicating weak (light gray) and moderate (darker gray) associations based on degrees of freedom (Statistical analysis and Table S2);

a *Results should be interpreted with caution because >20% of cells had expected frequencies lower than n = 5*.

### Relations with patient-specific characteristics (*n* = 286)

Topographical classification related moderately with the pelvic patterns in the transverse plane (*p* < 0.0001) and coronal plane (*p* < 0.0001) as well as with the knee patterns during swing (*p* < 0.0001) in the sagittal plane (Figure 1). Patients with unilateral CP were observed more often than expected with “excessive pelvic external rotation,” “pelvic depression,” and “minor gait deviations” in the knee during swing phase. In addition, patients with bilateral CP were classified more often with “increased pelvic range of motion” in the transverse plane, and “delayed peak knee flexion” during swing.

Age showed moderate associations with the knee patterns during swing (*p* < 0.0001) and ankle patterns during stance (*p* < 0.001) in the sagittal plane (Figure 2). A “horizontal” or “reversed second ankle rocker” was observed significantly more often in the youngest patients, whereas the oldest patients were more often classified as “calcaneus gait” or with “minor gait deviations.” The youngest patients also showed more often a “delayed peak knee flexion” or a “delayed and increased peak knee flexion” during swing.

GMFCS level was moderately associated with the patterns of the pelvis (*p* < 0.0001) and hip (*p* < 0.0001) in the sagittal plane (Figure 3). Moderate associations were also found for the knee during stance and swing, as well as the ankle during stance. However, the results of these χ^2^ analyses should be interpreted with caution due to the low number of patients classified as GMFCS level III in combination with pathological patterns that showed a low prevalence [e.g., equinus gait (4.9%)]. In general, patients with GMFCS level I were observed significantly more often in the patterns with “minor gait deviations” for the pelvis, hip, knee, and ankle joints in the sagittal plane. Patients with GMFCS levels II and III also displayed the patterns “hip extension deficit” and “increased pelvic anterior tilt” significantly more often than expected.

### Relations with side-specific variables and clinical symptoms (*n* = 446)

Previous surgery was moderately associated with the ankle patterns during swing (*p* < 0.0001; Figure 4). The categories that mainly contributed to this association were the higher frequency of “excessive dorsiflexion during swing” in combination with limbs that had undergone previous surgery.

The hip in the coronal plane was the only joint not associated with weakness or spasticity (Table 5). Further, only weak associations were identified for all joints in the coronal and transverse plane. Even though the associations were all weak, it was notable that the pattern “excessive hip internal rotation” was observed significantly more often in combination with higher levels of spasticity (MAS 2, 3, or 4) and weakness (MMT 0, 1, 2, or 3) for the muscles acting around the hip, knee, and ankle (Table S7).

In the sagittal plane, spasticity scores for muscles around the hip were moderately associated with the pelvis and hip patterns in the sagittal plane (*p* < 0.0001). Weakness at the level of the hip was moderately associated with the sagittal pelvis patterns (*p* < 0.0001), and weakly associated with the sagittal hip patterns (*p* < 0.0001; Figure 5 and Video 1). The pattern with “minor gait deviations” in both the pelvis and hip joints was observed significantly more often in limbs with few signs of spasticity (MAS scores 0, 1) or weakness (MMT scores 4, 5). On the other hand, pathological patterns such as “increased pelvic anterior tilt and increased range of motion” or “continuous excessive hip flexion” were mainly observed in limbs that were markedly affected by spasticity (MAS 1+, 2, 3, 4) or weakness (MMT 0, 1, 2, 3).

Severity of spasticity around the knee joint was moderately associated with the knee patterns both during stance and swing (*p* < 0.0001; Figure 6 and Video 1). A moderate association was also identified between weakness scores at the level of the knee and the knee patterns during swing (*p* < 0.0001). For the knee patterns during swing, it was apparent that all patterns with the feature “delayed peak knee flexion” (KSWS1, KSWS3, KSWS5; Figure 6 and Video 1) were observed significantly more often in combination with higher levels of spasticity (MAS 2, 3, 4) and weakness (MMT 0, 1, 2, 3). For the knee patterns during stance, “minor gait deviations” and “increased knee flexion at initial contact” were mainly observed in limbs with few signs of spasticity (MAS 0, 1) or weakness (MMT 4, 5). Limbs with higher levels of spasticity (MAS 2, 3, 4) or weakness (MMT 0, 1, 2, 3) were classified more often than expected as “increased knee flexion at initial contact and knee hyperextension” as well as “increased flexion during midstance and internal flexion moment present.”

Spasticity at the level of the ankle was moderately associated with the ankle patterns during stance (*p* < 0.0001; Figure 4 and Video 1), and weakly associated with the ankle patterns during swing (*p* = 0.001). The patterns “equinus gait” and “reversed second ankle rocker” were mainly observed in combination with marked signs of spasticity (MAS 2, 3, 4). Weakness at the level of the ankle was weakly associated with the ankle patterns both during stance and swing (both *p* < 0.01).

## Discussion

In this exploratory study, the prevalence of joint patterns during gait in children with CP and their association to patient-specific characteristics, previous surgery, and clinical symptoms, was examined.

The pattern “minor gait deviations” was observed most frequently in all joints, apart from the knee during stance and the pelvis in the sagittal plane. The prevalence of “minor gait deviations” reached more than 50% for the hip across the three anatomical planes, the pelvis in the coronal plane, and the foot progression angle. The need to define a pattern showing mild gait pathology has also been reported before, for example for the classifications of Winters et al. (1987) (hemiplegic patterns) and Rodda et al. (2004) (diplegic patterns) (Riad et al., 2007; McDowell et al., 2008; de Morais Filho et al., 2014). In both population- and hospital-based recruitment settings, the prevalence of these mild patterns has been reported to range between 12–43% (McDowell et al., 2008; de Morais Filho et al., 2014). The numbers in this study are generally higher, but this may be explained by the fact that the gait patterns in this study were evaluated at joint level, in contrast to the previous studies where total gait patterns, including multiple joints, have been reported. In the present study, however, a high number of “minor gait deviations” in specific joints does not imply that most children with CP in this study walked closely to typical gait in general. Indeed, it was found that at patient level, only 6.7% of the included limbs were classified with “minor gait deviations” in at least eight joints (out of 11 joints spread over the three anatomical planes), indicating that gait is markedly pathological in the majority of patients. So far, the way in which the various joint patterns across different planes combine in a total gait pattern is not yet fully understood.

Comparison of the prevalence of the pathological patterns to results from previous research is very challenging, as definitions of gait patterns as well as recruitment methods and inclusion criteria vary substantially across studies. For example, observed frequencies of excessive pelvic or hip rotation or in/outtoeing were markedly lower than the frequencies reported in previous studies (Wren et al., 2005; O'Sullivan et al., 2007; de Morais Filho et al., 2009; Simon et al., 2015). However, the definition of what constitutes excessive rotation across studies varies substantially. In the present study, a more strict definition was used by evaluating excessive rotation continuously over the entire gait cycle (or stance phase for FPA). This strict criterion is justified, taking into account the previously reported higher measurement errors for hip rotation and FPA (Schwartz et al., 2004). A notable finding of the current study was that both patterns showing “decreased pelvic anterior tilt” (or posterior tilt) with or without increased range of motion were observed only four times. Posterior pelvic tilt was previously included as a potential feature of the type IV gait pattern defined by Rodda et al. (2004), although it is unclear how often this feature is present in patients with type IV gait pattern (Rodda et al., 2004; Stott et al., 2005). The type IV pattern is mainly described for severely affected children. Following the assumption that posterior tilt will therefore be more prevalent in children with fewer functional abilities, the present study might have underestimated the prevalence of this pattern due to the relatively smaller sample size of children with GMFCS level III. Interestingly, the results of the previously mentioned study on the content validity of the Delphi gait classification (Nieuwenhuys et al., 2017a) indicated that all patterns, apart from PS4 and PS5, were statistically different from each other and from the patterns of TD children. With the low frequencies observed for the patterns PS4 and PS5 in the current study, and taking into consideration that the recent content validity study (Nieuwenhuys et al., 2017a) did not identify significant differences between these two joint patterns, the relevance of including both features as separate patterns in the classification should be re-examined in future research.

### Relations with patient-specific characteristics and clinical symptoms

It was hypothesized that the prevalence of the patterns would be associated with age, topographical classification, and GMFCS level. This hypothesis could be confirmed for some joints, but the strength of most identified associations was weak. The knee patterns during swing and the pelvis patterns in the frontal and transverse plane showed moderate associations with topographical classification. Hence, they can be considered as characterizing for children with unilateral or bilateral CP. The finding that children with unilateral CP have a relatively higher prevalence of pelvic depression and excessive pelvic external rotation compared to children with bilateral CP concurs with previous research investigating hemiplegic gait (Graham et al., 2005; O'Sullivan et al., 2007; Salazar-Torres et al., 2011). The results further showed that the prevalence of the ankle patterns during stance associated moderately with age, with the youngest patients showing a relatively higher frequency of a horizontal or reversed second ankle rocker. Wren et al. (2005) also noted decreased odds of equinus and increased odds of calcaneus gait with increasing age. The definition of equinus in their study (i.e., ankle plantarflexion >1 standard deviation below the mean for normal gait), would include the horizontal and reversed second ankle rocker, as well as the equinus pattern from the present study. These authors also reported an increased likelihood of presenting with internal hip rotation and/or outtoeing with increasing age (Wren et al., 2005). The present study also found that intoeing occurred significantly less often than expected in older subjects, but no significant association was identified between hip patterns in the transverse plane and age. Different definitions of excessive internal hip rotation between both studies might again be the main cause of the marked differences in the observed frequency of this pattern (ca. 40% in Wren et al., 2005, vs. 16.6% in this study). GMFCS levels are best characterized by the joint patterns in the sagittal plane. Although the results for the ankle and knee patterns should be interpreted with caution, a trend showed that patterns with minor gait deviations at the level of each joint were mainly observed in children with GMFCS I.

The study also examined how specific joint patterns during gait were characterized by weakness and spasticity. An obvious trend regarding all significant associations was that the patterns with minor gait deviations (PS0, HS0, KSTS0, KSWS0, ASTS0, ASWS0, PC0, PT0, HT0, FT0) were observed significantly more often in limbs with a low level of spasticity (MAS 0, 1, 1+) and good muscle strength (MMT 4 or 5), which appeared significantly less often than expected in other pathological patterns. The pathological patterns that were most characterized by both weakness (MMT 0, 1, 2, or 3) and spasticity were patterns related to pelvic anterior tilt (PS2 and PS3), patterns with increased knee flexion at initial contact (KSTS1 and KSTS4), patterns with abnormal knee flexion in swing (KSWS1, KSWS2, and KSWS5), ankle patterns characterized by excessive plantar flexion (ASTS3 and ASWS2), and “excessive hip internal rotation” (HT2). The patterns “increased and delayed peak knee flexion during swing” (KSWS3) and “outtoeing” (FPA1) were mainly characterized by weakness alone. On the other hand, “reversed second ankle rocker” (ASTS2) and “intoeing”' (FPA2) were mainly characterized by spasticity. It was also apparent that stronger associations with clinical symptoms were consistently found for the joints in the sagittal plane, possibly because most of the evaluated muscles in this study also perform sagittal plane motions as a main function (i.e., flexion and extension around the hip, knee, and ankle). Some of these associations are demonstrated in Video 1, where video fragments of patients' gait are provided as additional support to the kinematic waveforms and the respective joint gait patterns.

Remarkably, there were no significant associations identified with any of the investigated variables for the hip in the coronal plane. A recent study evaluated the level of clinician agreement with which these patterns could be identified and found that the hip in the coronal plane had the highest number of “unclassifiable” patients (Nieuwenhuys et al., 2017b). A future point of attention could be to investigate whether deviations in the coronal plane are compensations for deviations in the sagittal or transverse plane, as suggested by Davids et al. (Davids and Bagley, 2014). Hence, the pattern definitions of the coronal plane patterns and their relevance or necessity in the classification could be re-examined.

Another hypothesis said that a specific joint would be associated in particular with the severity of weakness or spasticity in muscle groups that act around that joint. The results of this study confirmed that these associations were present, however, as Tables 4, 5 demonstrate, joint patterns were also associated with weakness and spasticity scores of muscle groups acting around the other joints. For instance, for the knee patterns during swing, a significant association was found with the level of spasticity for the muscles around the knee, but also with the level of spasticity around the ankle and hip joint. The directions of these significant associations were the same for the spasticity scores at each level: with higher scores of spasticity, the patterns “delayed (and decreased)” peak knee flexion (KSWS1, KSWS5) were observed significantly more often; with lower scores of spasticity, the patterns “minor gait deviations” (KSWS0), and “increased peak knee flexion” (KSWS2) were more often observed. This finding can be extrapolated to all joint patterns: if joint patterns were associated with weakness or spasticity at more than one level (i.e., hip, knee, or ankle), the direction of the significant associations was similar for all levels (Tables S3–S7). This result suggests that specific gait deviations in one joint are not only caused by problems in the muscles surrounding that joint. They will rather be the result of a complex interplay of different muscles and movements at all lower limb joints.

## Limitations

A few limitations of the study need to be addressed. The generalizability of the results of this study might be limited as the investigated study group was a sample of convenience, recruited from one hospital setting. Firstly, it was noted that there was an underrepresentation of patients with GMFCS III and an overrepresentation of patients with unilateral cerebral palsy in the studied sample compared to previously reported distributions of gross motor function and topographical classifications (Gorter et al., 2004; Rosenbaum, 2008). More clear trends with GMFCS level might be identified given a larger proportion of children with GMFCS III, especially for the knee patterns and for the ankle patterns during stance. Secondly, 70 of 356 patients were excluded, of which 14 patients (20%) were excluded due to missing data from the clinical examination. It was not possible to find out the precise reasons for these missing data (e.g., fatigue or age resulting in reduced collaboration of the child, oversight by clinician, etc.). As a result, a small bias toward the exclusion of weaker or more severely affected children in the studied sample cannot be excluded. Thirdly, because the study used retrospective data, a relatively large amount of patients had undergone previous Achilles tendon lengthening (29 out of 100 limbs that were operated upon). The generalizability of the results is therefore limited, as surgical strategies have evolved during the past 10 to 20 years and tendon lengthening procedures are performed much less frequently (Gage, 2003; Healy et al., 2011). It is therefore difficult to formulate strong conclusions regarding the influence of previous surgery on the distribution of the joint patterns. In the future, the effect of previous surgery should be investigated using more specific subgroups regarding previous surgical interventions, or alternatively, prospective longitudinal intervention studies should be carried out to test the responsiveness of the patterns to different treatment interventions. Another limitation of this study is that due to the sample size, a comparison between males and females, especially in the older patient group (>12 years old) could not be performed. In addition, in patients who were bilaterally involved, for side-specific variables both sides were included in the statistical analyses, whereas for the remaining comparisons, only one trial from one side was randomly selected. In this way, the possible effect of the contralateral leg in the observed gait patterns was not taken into consideration in the performed analyses. In this study, it was further decided to group muscles at the level of each joint depending on their main function, and to select the most severe MAS or MMT score to represent the severity of spasticity or weakness at that joint. This implicates that when weakness at the level of the ankle is associated with specific ankle patterns, some of the scores used for statistical analysis might have been the result of ankle dorsiflexor weakness, others might have been due to ankle plantarflexor weakness. It is obvious that different muscles such as ankle plantar- and dorsiflexors would affect gait differently and potentially stronger associations might be discovered if these analyses would be performed on a muscle-specific rather than joint-specific basis. However, detailed investigations of the muscle-specific MMT and MAS scores around each joint revealed that problems of spasticity or weakness were mostly present in more than one muscle group. Because several muscles are affected by weakness or spasticity to a similar extent, and because different categories of the MAS and MMT scale were merged, it can be assumed that muscle-specific analyses would not change the general interpretations of the currently presented results. Rather, they might point to specific muscles whose clinical characteristics are discriminating best between particular joint patterns during gait. Because different categories of the MAS and MMT scale were merged, the potential bias that could result from the missing clinical data in the analyzed patient sample, was also further reduced. Lastly, the classifications for each limb were based on a single representative trial, whereas CP children are known to have a certain amount of variability across trials. Future research may evaluate to what extent this variability affects the classifications and how consistently these patterns are assigned across multiple trials.

## Conclusion

The usefulness of any classification essentially relies on its potential to make distinctions between clinically relevant subgroups in CP. This study provided first insights toward the construct validity and clinical relevance of joint gait patterns in CP (Nieuwenhuys et al., 2016). Although further validation is warranted, the results of this study confirm that most joint patterns during gait are characterized by different patient-specific characteristics and that they are often associated with gross categories of muscle weakness and spasticity.

## Author contributions

This study was designed by AN, EP, TD, and KD. AN and EP were responsible for all data acquisition and data analysis, SS created the Supplementary Material—Video 1. At each stage of the study, all authors have had complete access to the study data. Each author contributed to the interpretation of the results and was involved in the critical revision and editing of the manuscript that was written by AN and EP. All authors approve the final version of the manuscript and agree to be accountable for the content of the work.

## Funding

AN is supported by an OT project of KU Leuven University (OT/12/100). EP is supported by the MD Paedigree project, a Model-Driven pediatric European Digital Repository, partially funded by the European Commission under FP7—ICT Programme (grant agreement no: 600932, http://www.md-paedigree.eu) and by the SIMCP IWT-project, a simulation platform to predict gait performance following orthopedic intervention in children with cerebral palsy (IWT 140184). A grant from the Doctoral Scholarships Committee for International Collaboration with non-EER-countries (DBOF) of the KU Leuven, Belgium, was awarded to KD, grant number DBOF/12/058, for the PhD of SS. SS was further supported by an SBO grant from the Flemish Agency for Innovation by Science and Technology, IWT: grant 120057.

### Conflict of interest statement

The authors declare that the research was conducted in the absence of any commercial or financial relationships that could be construed as a potential conflict of interest.
