# Supplementary material for: Prevalence of Joint Gait Patterns Defined by a Delphi Consensus Study Is Related to Gross Motor Function, Topographical Classification, Weakness, and Spasticity, in Children with Cerebral Palsy
Source: Front Hum Neurosci. 2017 Apr 12;11:185. doi: 10.3389/fnhum.2017.00185 (PMC5388743; doi:10.3389/fnhum.2017.00185)
Supplement: Supplementary file 1 [file Table1.docx]

Supplementary Material

**Prevalence of joint gait patterns defined by a Delphi consensus study is related to gross motor function, topographical classification, weakness, and spasticity, in children with cerebral palsy**

**Angela Nieuwenhuys, Eirini Papageorgiou, Simon-Henri Schless, Tinne De Laet, Guy Molenaers, Kaat Desloovere***

*** Correspondence:** [kaat.desloovere@uzleuven.be](mailto:kaat.desloovere@uzleuven.be)

# Supporting Information Tables

| Table S1. Final overview of joint patterns and their criteria after last Delphi survey | | | |
| --- | --- | --- | --- |
|  |  | **Joint pattern** | **Full description and criteria** |
| **Sagittal plane** | **Pelvis** | Minor gait deviations* | Deviations within range of normal values |
|  |  | Increased range of motion | Range of motion, which is greater than normal values |
|  |  | Increased pelvic anterior tilt on average | Pelvic anterior tilt, which is greater than normal values |
|  |  | Increased pelvic anterior tilt + increased range of motion | Pelvic anterior tilt + range of motion, which are greater than normal values |
|  |  | Decreased pelvic tilt (posterior tilt) on average | Pelvic anterior tilt, which is lower than normal values |
|  |  | Decreased pelvic tilt (posterior tilt) + increased range of motion | Pelvic anterior tilt, which is lower + range of motion, which is greater than normal values |
|  | **Hip** | Minor gait deviations* | Deviations within range of normal values |
|  |  | Hip extension deficit | At least two of the following characteristics: (1) decreased hip extension in stance, (2) decreased hip range of motion in stance, (3), delayed timing of zero hip moment or decreased hip flexion moment |
|  |  | Continuous excessive hip flexion | Excessive hip flexion throughout at least 90% of the gait cycle AND hip flexion angle continuously above 0° |
|  | **Knee** | Minor gait deviations* | Deviations within range of normal values |
| **(during stance)** | | Increased knee flexion at initial contact | Knee flexion at initial contact, which is greater than normal values |
|  |  | Increased knee flexion at initial contact + earlier knee extension movement | Knee flexion at initial contact, which is greater + peak knee flexion angle, which is sooner than normal values |
|  |  | Knee hyperextension | At least two of the following characteristics: (1) increased knee extension (to knee hyperextension) in mid- or late stance, (2) earlier knee extension movement in stance, (3) excessive knee flexion moment in mid- or late stance |
|  |  | Knee hyperextension + increased knee flexion at initial contact | Increased knee flexion at initial contact AND at least 2 out of 3 features: (1) increased knee extension (to knee hyperextension) in mid- or late stance, (2) earlier knee extension movement in stance, (3) excessive knee flexion moment in mid- or late stance |
|  |  | Increased knee flexion in midstance + internal knee flexion moment present | Increased knee flexion in midstance: no normal knee angle in extension in midstance AND internal knee flexion moment is present for at least 1/3rd of stance phase |
|  |  | Increased knee flexion in midstance + internal knee extension moment present | Increased knee flexion in midstance: no normal knee angle in extension in midstance AND internal knee extension moment is present for at least 2/3rd of stance phase |
|  |  |  |  |
|  |  |  |  |
|  |  |  |  |
| (Table S1. Continued) | | | |
|  | **Knee** | Minor gait deviation* | Deviations within range of normal values |
| **(during swing)** | | Delayed peak knee flexion | Peak knee flexion during swing, which is more delayed than normal values |
|  |  | Increased peak knee flexion | Peak knee flexion during swing, which in greater than normal values |
|  |  | Increased + delayed peak knee flexion | Peak knee flexion during swing, which is greater and more delayed than normal values |
|  |  | Decreased peak knee flexion | Peak knee flexion during swing, which is lower than normal values |
|  |  | Decreased + delayed peak knee flexion | Peak knee flexion during swing, which is lower and more delayed than normal values |
|  | **Ankle** | Minor gait deviations* | Deviations within range of normal values |
| **(during stance)** | | Horizontal second ankle rocker | Horizontal pattern of second ankle rocker from loading response (10%) to start push-off (slope <5°) |
|  |  | Reversed second ankle rocker | Descending pattern of second ankle rocker from loading response (10%) to start push-off (slope ≥ -5°) |
|  |  | Equinus | Continuous plantarflexion (x<0°) throughout stance |
|  |  | Calcaneus gait | Increased slope towards dorsiflexion during stance OR a peak ≥ 20° of dorsiflexion |
|  | **Ankle** | Minor gait deviations* | Deviations within range of normal values |
| **(during swing)** | | Insufficient prepositioning in terminal swing | Ankle plantarflexion at initial contact at the end of the gait cycle, which is greater than normal values |
|  |  | Continuous plantarflexion in swing (drop foot) | Excessive plantarflexion for most of the swing phase, at least hindering foot clearance around 90% of the gait cycle AND ankle plantarflexion at initial contact at the end of the gait cycle, which is greater than normal values |
|  |  | Excessive dorsiflexion in swing | Increased dorsiflexion in swing for at least 1/3rd of the swing phase |
| **Coronal plane** | **Pelvis** | Minor gait deviations* | Deviations within range of normal values |
|  |  | Increased pelvic range of motion | Range of motion, which is greater than normal values |
|  |  | Continuous pelvic elevation (up) | Pelvic elevation, which is greater than normal values |
|  |  | Continuous pelvic depression (down) | Pelvic depression, which is greater than normal values |
|  | **Hip** | Minor gait deviations* | Deviations within range of normal values |
|  |  | Excessive hip abduction in swing | Hip abduction in swing, which is greater than normal values |
|  |  | Continuous excessive hip abduction | Hip abduction throughout gait cycle, which is greater than normal values |
|  |  | Continuous excessive hip adduction | Hip adduction throughout gait cycle, which is greater than normal values |
|  |  |  |  |
|  |  |  |  |
|  |  |  |  |
| (Table S1. Continued) | | |  |
| **Transverse plane** | **Pelvis** | Minor gait deviations* | Deviations within range of normal values |
|  |  | Increased pelvic range of motion | Range of motion, which is greater than normal values |
|  |  | Excessive pelvic external rotation during the gait cycle | Pelvic external rotation, which is greater than normal values |
|  |  | Excessive pelvic internal rotation during the gait cycle | Pelvic internal rotation, which is greater than normal values |
|  | **Hip** | Minor gait deviations* | Deviations within range of normal values |
|  |  | Excessive hip external rotation during the gait cycle | Hip external rotation, which is greater than normal values |
|  |  | Excessive hip internal rotation during the gait cycle | Hip internal rotation, which is greater than normal values |
|  | **Foot** | Minor gait deviations* | Deviations within range of normal values |
|  |  | Outtoeing | Excessive external foot progression on average during stance |
|  |  | Intoeing | Excessive internal foot progression on average during stance |

John Wiley and Sons, © 2015 Mac Keith Press; Reprinted and modified [1]. Modifications of the original definitions are indicated by a ‘*’.

[1] Nieuwenhuys A, Õunpuu S, Van Campenhout A, Theologis T, De Cat J, Stout J, et al. Identification of joint patterns during gait in children with cerebral palsy: A Delphi consensus study. Dev Med Child Neurol 2016;58:306–13. doi:10.1111/dmcn.12892.
