# Supplementary material for: Prevalence of Joint Gait Patterns Defined by a Delphi Consensus Study Is Related to Gross Motor Function, Topographical Classification, Weakness, and Spasticity, in Children with Cerebral Palsy
Source: Front Hum Neurosci. 2017 Apr 12;11:185. doi: 10.3389/fnhum.2017.00185 (PMC5388743; doi:10.3389/fnhum.2017.00185)
Supplement: Supplementary file 2 [file Table2.docx]

Supplementary Material

**Prevalence of joint gait patterns defined by a Delphi consensus study is related to gross motor function, topographical classification, weakness, and spasticity, in children with cerebral palsy**

**Angela Nieuwenhuys, Eirini Papageorgiou, Simon-Henri Schless, Tinne De Laet, Guy Molenaers, Kaat Desloovere***

*** Correspondence:** [kaat.desloovere@uzleuven.be](mailto:kaat.desloovere@uzleuven.be)

# Supporting Information Tables

| **Table S2.** Cut-off values to interpret the strength of a significant association between two variables using Cramer's V statistic are dependent on the degrees of freedom (DF) [1]. | | | | | |
| --- | --- | --- | --- | --- | --- |
|  |  |  |  |  |  |
|  |  |  |  |  |  |
|  | | **Cramer's V** | | **Interpretation** | |
| DF = 1 | | 0.10 < V < 0.30 | | Weak association | |
|  |  | 0.30 < V < 0.50 | | Moderate association | |
|  |  | V > 0.50 | | Strong association | |
|  |  |  |  |  |  |
| DF = 2 | | 0.07 < V < 0.21 | | Weak association | |
|  |  | 0.21 < V < 0.35 | | Moderate association | |
|  |  | V > 0.35 | | Strong association | |
|  |  |  |  |  |  |
| DF = 3 | | 0.06 < V < 0.17 | | Weak association | |
|  |  | 0.17 < V < 0.29 | | Moderate association | |
|  |  | V > 0.29 | | Strong association | |
| DF is the smaller value of (R-1) or (C-1). R and C represent the number of categories of the related variables. | | | | | |
|  |  |  |  |  |  |
|  |  |  |  |  |  |
