# Supplementary material for: Prevalence of Joint Gait Patterns Defined by a Delphi Consensus Study Is Related to Gross Motor Function, Topographical Classification, Weakness, and Spasticity, in Children with Cerebral Palsy
Source: Front Hum Neurosci. 2017 Apr 12;11:185. doi: 10.3389/fnhum.2017.00185 (PMC5388743; doi:10.3389/fnhum.2017.00185)
Supplement: Supplementary file 3 [file Table3.docx]

Supplementary Material

**Prevalence of joint gait patterns defined by a Delphi consensus study is related to gross motor function, topographical classification, weakness, and spasticity, in children with cerebral palsy**

**Angela Nieuwenhuys, Eirini Papageorgiou, Simon-Henri Schless, Tinne De Laet, Guy Molenaers, Kaat Desloovere***

*** Correspondence:** [kaat.desloovere@uzleuven.be](mailto:kaat.desloovere@uzleuven.be)

# Supporting Information Tables

Tables S3-S7 indicate the direction of the significant associations between all gait patterns and the different categories of patient-specific characteristics (N=286), side-specific, and clinical variables (N=446). Numbers in the table represent the significant adjusted standardized residuals and indicate the specific combinations of categories and patterns that were observed more often (positive values) or less often (negative values) than would be expected if the variables were unrelated. Detailed cross-tables including the observed frequencies and percentages of the recruited sample population are available at request with the authors. Pattern definitions are available in Table S1 in the online supporting information.
